# Mitophagy-Mediated mtDNA Release Aggravates Stretching-Induced Inflammation and Lung Epithelial Cell Injury via the TLR9/MyD88/NF-κB Pathway

**DOI:** 10.3389/fcell.2020.00819

**Published:** 2020-09-04

**Authors:** Ren Jing, Zhao-Kun Hu, Fei Lin, Sheng He, Sui-Sui Zhang, Wan-Yun Ge, Hui-jun Dai, Xue-Ke Du, Jin-Yuan Lin, Ling-Hui Pan

**Affiliations:** ^1^Department of Anesthesiology, Guangxi Medical University Affiliated Tumor Hospital & Oncology Medical College, Nanning, China; ^2^The Laboratory of Perioperative Medicine Research Center, Guangxi Medical University Affiliated Tumor Hospital & Oncology Medical College, Nanning, China

**Keywords:** mitophagy, mitochondrial DNA, Toll-like receptor 9, mechanical stretching, lung injury

## Abstract

**Background:**

In animal models of ventilation-induced lung injury, mitophagy triggers mitochondria damage and the release of mitochondrial (mt) DNA, which activates inflammation. However, the mechanism of this process is unclear.

**Methods:**

A model of cyclic stretching (CS)-induced lung epithelial cell injury was established. The genetic intervention of phosphatase and tensin homolog-induced kinase 1 (PINK1) expression via lentivirus transfection was used to identify the relationship between PINK1-mediated mitophagy and mtDNA release in stretching-induced inflammatory response and injury. Pharmacological inhabitation of Toll-like receptor 9 (TLR9) and myeloid differentiation factor 88 (MyD88) expression was performed via their related inhibitors, while pre-treatment of exogenous mtDNA was used to verify the role of mtDNA in stretching-induced inflammatory response and injury.

**Results:**

Using a cell culture model of CS, we found that knocking down PINK1 in lung epithelial cells reduced mitophagy activation and mtDNA release, leading to milder inflammatory response and injury; conversely, up-regulating PINK1 exacerbated stretching-induced inflammation and injury, and similar effects were observed by upregulating TLR9 to induce expression of MyD88 and nuclear factor-κB (NF-κB)/p65. Down-regulating MyD88 protected lung epithelial cells from stretching injury and decreased NF-κB/p65 expression.

**Conclusion:**

These findings suggest that PINK1-dependent mitophagy and associated TLR9 activation is indeed a major factor in stretch-induced cell injury via a mechanism in which released mtDNA activates TLR9 and thereby the MyD88/NF-κB pathway. Inhibiting this process may be a therapeutic approach to prevent inflammation and cell injury in patients on mechanical ventilation.

## Introduction

Mechanical ventilation with high tidal volume, often used for patients with acute respiratory distress syndrome or severe pneumonia, causes so-called ventilator-induced lung injury (VILI) (Vieillard-Baron and Dreyfuss, 2017), in an estimated 24% of patients (Gajic et al., 2004). VILI involves increasing pulmonary and vascular permeability, infiltration by inflammatory cells, inflammation, and oxidative stress (Papaiahgari et al., 2007; Christaki, 2009; Gu et al., 2015), as well as the robust release of pro-inflammatory cytokines and signaling pathways (Del Sorbo and Slutsky, 2011).

Using a rat model of mechanical ventilation with high tidal volume, we showed that cyclic stretching (CS) triggered mitophagy, which led to the release of mitochondrial (mt) DNA. The mtDNA acted as a damage-associated molecular pattern (DAMP) that was recognized by Toll-like receptor (TLR) 9, activating the TLR9/myeloid differentiation factor 88 (MyD88)/nuclear factor-κB (NF-κB) signaling pathway, ultimately aggravating inflammation and lung injury (Dai et al., 2015; Huang et al., 2017; Jing and Pan, 2017).

Phosphatase and tensin homolog induced kinase 1 (PINK1) is ubiquitously expressed, which plays an important role in the maintenance of mitochondrial morphology and function as well as in the selective degradation of damaged mitochondria by mitophagy (Unoki and Nakamura, 2001; Di Rita et al., 2018; Matheoud et al., 2019). *Pink1* gene mutations are associated with mitochondrial quality control differently according to cell age, type, and stress levels, suggesting that metabolic capacity and adaptation affect cellular vulnerability to PINK1 deficiency (Billia et al., 2011). PINK1 deficiency would trigger dysfunctional mitochondria and defective mitophagy and promotes fibrosis in the aging lung (Bueno et al., 2015).

To continue to elucidate the molecular mechanisms by which released mtDNA stimulates stretching-induced inflammation and injury, we explored here whether PINK1 may help induce the mitophagy that causes inflammation and injury in VILI. As a vitro model of VILI, we subjected human alveolar type II A549 cells to CS.

## Materials and Methods

### Reagents and Antibodies

Enzyme-linked immunosorbent assays (ELISAs) to assay interleukin-1β (IL-1β, catalog no. 70-EK101B1), IL-6 (catalog no. 70-EK1061), and tumor necrosis factor-alpha (TNF-α, catalog no. 70-EK1821) were purchased from Multi Sciences (Hangzhou, China). Dulbecco’s modified Eagle’s medium (DMEM) was purchased from Gibco (catalog no. 12100-46; Life Technologies, Waltham, MA, United States); fetal bovine serum (FBS), from Hyclone (catalog no. SH30084.03; Logan, UT, United States); oligodeoxyribonucleotides (ODN) 2088, from Miltenyi Biotec (catalog no. 130-105-815; Cologne, Germany); and ODN1668, from Alexis (catalog no. ALX-746-051-M001; Lausen, Switzerland). Pancreatin (catalog no. T4799), MTT Cell Proliferation and Cytotoxicity Assay Kit (catalog no. V13154), Mitochondrial Membrane Potential (ΔψM) Apoptosis Kit (catalog no. V35116), and adenosine triphosphate (ATP) Determination Kit (catalog no. A22066) were purchased from Sigma-Aldrich (St. Louis, MO, United States). ST2825 was purchased from MedChemExpress (catalog no. HY-50937; Monmouth Junction, NJ, United States); the Premo^TM^ Autophagy Tandem Sensor RFP-GFP-LC3B Kit, from Thermo Fisher Scientific (catalog no. P36239; Waltham, MA, United States); Mitochondria DNA Isolation Kit (BioVision, catalog no. K280), TRIzol (Invitrogen, catalog no. 15596018) and the Bicinchoninic Acid Protein Assay Kit (Pierce, catalog no. BCA1-1KT), from Thermo Fisher Scientific (Hilden, Germany). The PrimeScript^TM^ II 1st Strand cDNA Synthesis Kit (catalog no. 6210A) and PrimeScript^TM^ RT reagent Kit with gDNA Eraser (Perfect Real Time) (catalog no. RR047A) were purchased from Takara (Beijing, China).

Antibodies against the following proteins were purchased from Sigma-Aldrich (St. Louis, MO, United States): microtubule associated protein 1 light chain 3 β (MAP1LC3B, catalog no. L7543; used at 1: 1,000 dilutions), sequestosome 1 (SQSTM1, catalog no. P0067; 1:1000), parkin RBR E3 ubiquitin protein ligase (PRKN, catalog no. SAB4502077; 1:800), beclin 1 (BECN1, catalog no. HPA028249; 0.4 μg/ml), and dynamin 1 like (DNM1L, catalog no. HPA039324; 0.7 μg/ml). Antibodies against the following proteins were obtained from Novus (Centennial, CO, United States): PINK1 (catalog no. BC100-494; 1: 1,000) and TLR9 (catalog no. NBP1-76680; 1:1,000). Antibodies against the following proteins were bought from Cell Signaling Technology (Danvers, MA, United States): MyD88 (catalog no. 4283; 1: 1,000), BCL2 apoptosis regulator (Bcl-2, catalog no. 4223; 1:1, 000), BCL2-associated X apoptosis regulator (Bax, catalog no. 5023; 1:1,000), cleaved caspase3 (catalog no. 9664; 1:1,000), NF-κB/p65 (catalog no. 4764; 1:1,200), and ACTB (β-actin, catalog no. 4970; 1:1,000). Horseradish peroxidase (HRP)-conjugated mouse anti-rabbit secondary antibody was also purchased from Cell Signaling Technology (catalog no. 7074; 1:1,000).

### Cell Culture

Human alveolar basal epithelial cells (A549 cell lines) were purchased from Shanghai Zhongqiao Xinzhou Biotechnology (catalog no. ZQ0003; Shanghai, China). A549 cells were cultured at a density of 1 × 105 cells/cm^2^ in a complete medium consisting of DMEM supplemented with 10% (w/v) FBS in collagen IV-coated flexible-bottom BioFlex plates (Flexcell International, United States). Cells were grown at 37°C with pH 7.4 and 5% CO_2_ in air, until they reached 85% confluence. Previous 85% confluent monolayers formed on Flexible bottom BioFlex plates with elastomer membranes within 24–48 h, at which point A549 monolayers were serum-deprived for 2.0 h before CS experiments.

### Lentiviral Infection and Transfection With Small Interfering RNA (siRNA)

A549 cells were cultured with 90% cell density in each medium-sized cell culture bottle, harvested by trypsinization, centrifuged, resuspended in DMEM medium, and transferred to 15 ml cuvettes. The supernatant was discarded and cells were resuspended in 1.0 ml complete medium. The cells were seeded into 6-well plates at a density of 4 × 10^5^ per well in DMEM plus 10% FBS at 37°C with pH 7.4 and 5% CO_2_ in air.

*Pink1* siRNA (sc-44599), and scrambled siRNA (sc-37007) were obtained from Santa Cruz Biotechnology (Dallas, TX, United States). The full ORF cDNA clone of human PINK1 was purchased from ATCC catalog number 10627756, GenBank # AL391357.1. PINK1 was subcloned into the pCMV-3 (MYC)Taq-2 vector (Agilent Technologies, Cat#240196) by using restriction endonucleases EcoRI and XhoI (named as pCMV -MYC -PINK1). The virus was stored at 4°C and lightly shaken to transfect prepared cells with an appropriate concentration according to the manufacturer’s protocol. According to the gene sequence of human PINK1 (NM_032409.2, CDs region 1746bp), the lentiviral vector of *Pink1* cDNA over-expression, *Pink1* siRNA silencing, and their negative control were transfected into A549 cells, respectively. After 8–12 h of transfection, the target cells were washed and resuspended using a medium without a lentiviral vector.

The expression of target protein PINK1 was assessed by Western blot and real time-quantitative PCR (RT-qPCR), and transfection efficiency was assessed using fluorescence microscopy (Olympus). The subsequent experiments could be performed at 48–72 h after transfection.

### VILI Model *in vitro*

A549 cell monolayers in BioFlex plates were exposed to CS using the FX 5000T Flexercell Tension Plus system (Flexcell International, McKeesport, PA, United States) at a frequency of 30 cycles/min (0.5 Hz) and stretch/relaxation ratio of 1:1 (Wang et al., 2011). Based on previous studies (Carta et al., 2011; Wang et al., 2011), CS was conducted with a change of the basement membrane surface area by 8, 15, or 20% cyclically. These surface area changes correspond to 50%, 64%, and 80% of total lung capacities (Klionsky et al., 2016). In this model system, we used 5% tension as physiological stretching, and 20% tension as overstretching. CS was performed under computer control for 4.0 h at 37°C in a humidified incubator containing 5% CO_2_ in the air. Control cultures, derived from the same parental culture as the stretched cultures, were not subjected to stretching. Cells were cyclically stretched for 4.0 h or as indicated.

### Preparation and Treatments of mtDNA

The mtDNA from A549 cells undergoing 20% overstretching for 4.0 h was isolated using the Mitochondrial DNA Isolation Kit (BioVision, United States) following the manufacturer’s instructions. The concentration of mtDNA was measured by spectrophotometry, then the mtDNA was diluted to 1.0 μg/μl and immediately stored at −80°C. A549 cells were, respectively, treated with isolated mtDNA (1.0 μg/μl) or an equal volume of phosphate-buffered saline (PBS) (10^5^ cells per treatment) at the first 24 h of CS.

### Regulation of TLR9 and MyD88 Expression

A549 cells (10^5^ per treatment) were pretreated with TLR9 receptor antagonist (ODN2088, 0.1 μM) for 24 h, TLR9 receptor agonist (ODN1668, 2.5 μM) for 6 h, or MyD88 inhibitor (ST2825, 30 μM) for 48 h (Liang et al., 2011; Nadorp and Soreq, 2015; Shiratori et al., 2017). These various cultures were then subjected to CS.

### Autophagy Flows and Mitochondrial Detection

A549 cells were transfected with the Autophagy Tandem Sensor RFP-GFP-LC3B kit for 24 h according to the manufacturer’s protocol, then exposed to over-stretching or physiological stretching for 4.0 h. Autophagy flows were observed using fluorescence microscopy (Olympus) before CS (time1), immediately after CS (time2), and again 4.0 h after the end of CS (time3).

### Assessment of Inflammation and Cell Injury

After A549 cell lines were mechanically stretched, the culture medium was collected and centrifuged at 1000×*g* for 3 min, then the supernatant was frozen at −80°C. The supernatant was assayed for IL-1β, IL-6, and TNF-α levels using ELISAs according to the manufacturer’s instructions. The cellular injury was determined by measuring the level of apoptosis and cell viability. Apoptotic-related proteins including Bcl-2, Bax, and cleaved Caspase 3 were assessed by Western blot, and MTT assay was used to examine the viability of cells. Simultaneously, normal and stretched cells were prepared for transmission electron microscopy to observe cell injury ultrastructurally.

### Measurement of ΔψM and ATP

The ΔψM was determined using the JC-1 probe in the assay kit as our previous study (Lin et al., 2018) described. Briefly, on the day of assay, the control or stretched cells were incubated with 500 nM JC-1 according to the manufacturer’s instructions. Fluorescence was visualized using fluorescence microscopy (Olympus) with constant parameters. The area occupied by mitochondria in red fluorescence vs. green fluorescence per cell was calculated using the Image J software (version 1.50i). Further, to reflect mitochondrial function, ATP production was detected using a luciferase-based ATP assay kit according to the manufacturer’s instructions.

### Relative Quantification of mtDNA-79 and mtDNA-230 Copies

Quantitative analysis of mtDNA fragments was performed by qPCR as previously published (Budnik et al., 2013). Two primer sets specific for the mitochondrial ribosomal 16SRNA contains a 79-bp fragment (mtDNA-79) that includes DNA released by apoptotic cells and a 230-bp fragment (mtDNA-230) that corresponds to mtDNA released by non-apoptotic types of cell death. The sequence of the forward primer for both mtDNA fragments was 5′-CAGCCGCTATTAAAGGTTCG-3′. The sequence of the reverse primer for mtDNA-79 was 5′-CCTGGATTACTCCGGTCTGA-3′, and the reverse primer sequence for mtDNA-230 was 5′-GGGCTCTGCCATCTTAACAA-3′. Each 10-ml reaction system consisted of 1.0 ml DNA, 5.0 ml iQ SYBR Green Supermix (Takara), and 0.25 ml forward/reverse primer. Each run included water blanks as a negative control. PCR proceeded at 95°C for 15 min, followed by 40 cycles at 95°C for 15 s, 60°C for 30 s, and 72°C for 30 s. For melting curve analysis (to confirm the specificity of the PCR products), the final cycle was added with 95°C for 15 s, 60°C for 15 s, and 95°C for 15 s. The data were calculated by the 2^–ΔΔ*Ct*^ method, which evaluates mtDNA fragments relative to the housekeeping gene (ACTB) expression. All experiments were repeated in triplicate.

### Real-Time qPCR Analyses

Total RNA was isolated from A549 cells using a Trizol reagent according to the standard protocol. RNA concentration and purity were examined by spectrophotometry. Then cDNA was synthesized using the PrimeScript^TM^ II 1st Strand cDNA Synthesis Kit. The primer sequences for ACTB, PINK1, PRKN, DNM1L, LC3B-II, BECN1, SQSTM1, TLR9, MyD88, and NF-κB/p65 are shown in Table 1. RT-qPCR was carried out using the PrimeScript^TM^ RT Reagent Kit with gDNA Eraser (Perfect Real Time), according to the manufacturer’s instructions. The threshold amplification cycle number was determined for each reaction within the linear phase of the amplification plot, and relative gene expression was determined using the 2^–ΔΔ*Ct*^ method and housekeeping gene (ACTB) expression as the internal reference.

**TABLE 1:** Primer sequences used to detect target mRNAs.

**Gene**	**Primer sequence (5′→3′)**	**Product size (bp)**
PINK1	F: GACCTGAAATCCGACAACATCC	160
	R: CCATCAGACAGCCGTTTCC	
PRKN	F: CTGATCGCAACAAATAGTCGG	185
	R: CAAGGCAGGGAGTAGCCAAGT	
DNM1L	F: GAATGACCAAGGTGCTGTAG	124
	R: AGCTAGGGTTCTGCGACCAT	
LC3B	F: GAGCAGCATCCAACCAAA	215
	R: GAGATTGGTGTGGAGACGCT	
BECN1	F: CAAGATCCTGGACCGTGTCA	102
	R: TGGCACTTTCTGTGGACATCA	
SQSTM1	F: GCACCCCAATGTGATCTGC	168
	R: CGCTACACAAGTCGTAGTCTGG	
TLR9	F: CCGTGCAGCCGGAGATGTTT	171
	R: CCGTGAATGAGTGCTCGTGGTAG	
MyD88	F: AACTGGAACAGACAAACTATCG	154
	R: GAGACAACCACCACCATCC	
NF-κB	F: ATCCTGAAGGCTACCAACTA	179
	R: GACACCAGGTCAGGATTTTG	
ACTB	F: CTTAGTTGCGTTACACCCTTTCTTG	156
	R: CTGTCACCTTCACCGTTCCAGTTT	

*F, forward primer; R, reverse primer; PINK1, phosphatase and tensin homolog (PTEN) inducing putative kinase 1; PRK1, Parkin; DNM1L, dynamin 1 like; LC3B, microtubule associated protein 1 light chain 3 β; BECN, beclin 1; SQSTM1, sequestosome 1; TLR, Toll-like receptor; MyD88, myeloid differentiation factor 88; NF-κB, nuclear factor-κB; ACTB, β-actin.*

### Western Blotting Analysis

Total proteins were extracted from A549 cells and centrifuged. Then protein concentration in the supernatant was estimated using the BCA Protein Assay Kit. The supernatant was fractionated by SDS-polyacrylamide electrophoresis, and the proteins were transferred onto polyvinylidene difluoride membranes and blocked with 5% skim milk for 1–2 h. Subsequently, membranes were incubated with primary antibodies, followed by HRP-conjugated secondary antibody. Membranes were washed, and protein bands were detected by chemiluminescence using the ECL system. Relative band densities of the various proteins were measured from scanned films using Image Lab software (Version 5.0 Build 18; Bio-Rad Laboratories, Hercules, CA, United States).

### Statistical Analysis

Data were analyzed using the SPSS 22.0 software (IBM, Chicago, IL, United States). All quantitative data were reported as mean ± SD. Inter-group differences were evaluated for significance using one-way ANOVA and LSD-*t* tests. *P* < 0.05 was considered a significant statistical difference.

## Results

### Overstretching of Lung Epithelial Cells Activates Mitophagy and mtDNA Release

After 20% tension CS, cells showed obvious injury by transmission electron microscopy (Supplementary Figure S1A), and they secreted higher levels of IL-1β, IL-6, and TNF-α into the culture medium than when at 5% tension CS or not stretched at all (Supplementary Figures S1B–D). Then, cells under CS at 20% tension dramatically decreased cell viability (Supplementary Figure S1E), increased cell apoptosis (Supplementary Figure S1F), and reduced ATP levels (Supplementary Figure S1G) and ΔψM (Supplementary Figure S1HH) when compared with controlled cells or cells after CS at 5% tension.

**FIGURE 1:**
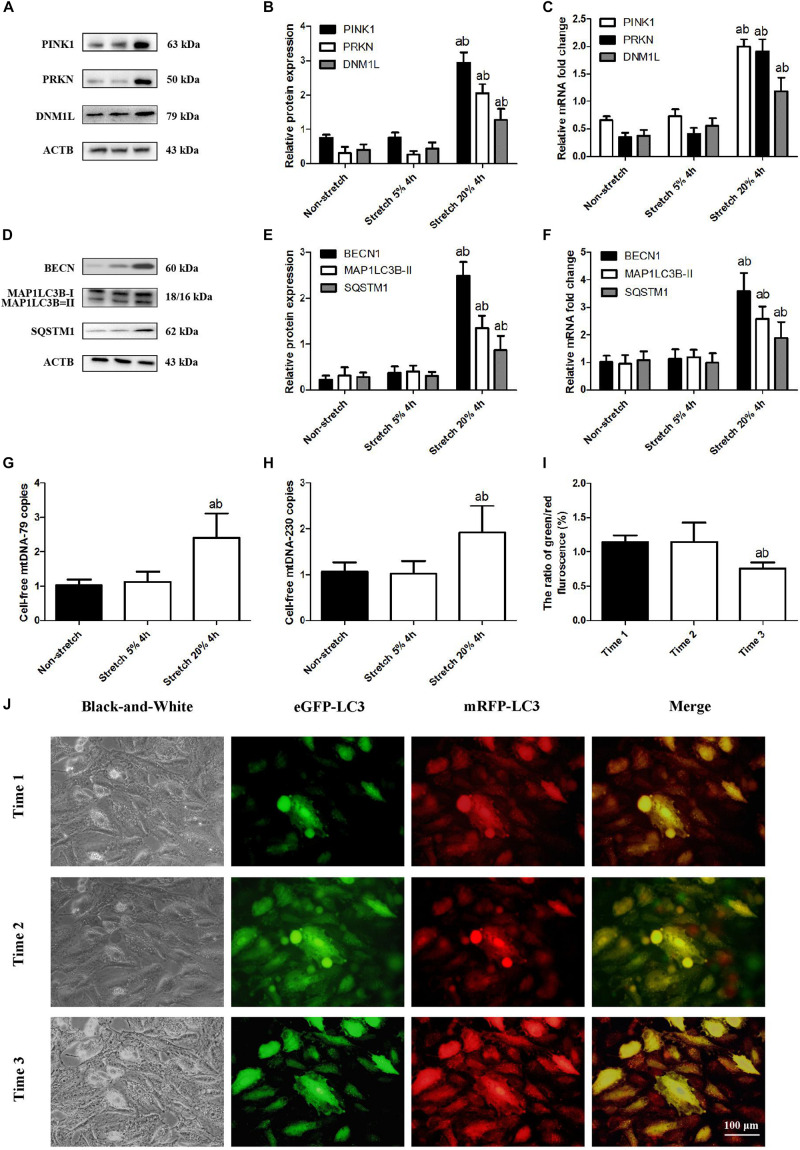
Overstretching of lung epithelial cells activates mitophagy and mtDNA release. Lung epithelial cells were challenged for 4 h with cyclic stretching (CS) at 5% or 20% tension or left without CS. **(A,B)** Western blotting was performed to determine the protein expression of PINK1, PRKN, and DNM1L. **(C)** RT-qPCR was simultaneously performed to assess mRNA levels of PINK1, PRKN, and DNM1L. **(D,E)** Western blotting was performed to determine the protein expression of BECN1, MAP1LC3B, and SQSTM1. **(F)** RT-qPCR was simultaneously performed to assess mRNA levels of BECN1, MAP1LC3B, and SQSTM1. **(G)** RT-qPCR was simultaneously performed to assess the cell-free mtDNA-79 copies. **(H)** RT-qPCR was simultaneously performed to assess the cell-free mtDNA-230 copies. **(I,J)** Quantitative analysis and observation of autophagy flux in A549 cells exposed to CS at 20% tension with the Autophagy Tandem Sensor RFP-GFP-LC3B Kit before 20% cyclic stretching (time1), immediately after cyclic stretching (time2), and again 4.0 h after the end of stretching (time3) by fluorescence microscopy (magnification ×400). Experiments were performed in triplicate. ^a^*P* < 0.05, compared with the lung epithelial cells without CS; ^b^*P* < 0.05, compared with the lung epithelial cells exposed to CS at 5% tension. **(I)** Quantitative analysis of autophagy flux. ^a^*P* < 0.05, compared with time1; ^b^*P* < 0.05, compared with time2.

Mitophagy is a two-step process by which the damaged mitochondria are first primed by PINK1/PRKN proteins and then eliminated via the autophagy. We investigated the expression of mitophagy-related proteins including PINK1, PRKN, and DNM1L in A549 lung epithelial cells at tensions of 5% or 20% CS. As shown in Figures 1A–C, levels of PINK1, PRKN, and DNM1L protein and mRNA were up-regulated in A549 cells after CS at 20% tension. We also examined the expression levels of such autophagy-related proteins as BECN1, MAP1LC3B-II (an autophagosome initiation marker), and SQSTM1 (an autophagosome formation marker). As shown in Figures 1D–F, in A549 cells exposed to CS at 20% tension, there was a significant up-regulation in the expression levels of the autophagy markers, BECN1, MAP1LC3B-II, and SQSTM1.

Previous work in rats suggested that VILI damages mitochondria primarily through mitophagy, such that mitophagy-released mtDNA makes up a significant fraction of total mtDNA (Lin et al., 2018). Consistent with this idea, we found that A549 cells after CS at 20% tension up-regulated the level of mtDNA-79 and mtDNA-230 (Figures 1G,H). These expression changes were also related to an increase in autophagic flux (Figures 1I,J). It suggested that A549 cells before CS did not activate autophagy; A549 cells immediately after CS induced autophagy with autophagosomes; A549 cells at 4.0 h after the end of CS showed the advanced autophagy with autolysosomes. Our results suggested that our cell culture model of CS recapitulates the mitophagy-induced release of mtDNA that we observed in our rat model of mechanical ventilation (Lin et al., 2018).

### *Pink1* Knockdown in Lung Epithelial Cells Attenuates CS-Induced Inflammation and Injury

**FIGURE 2:**
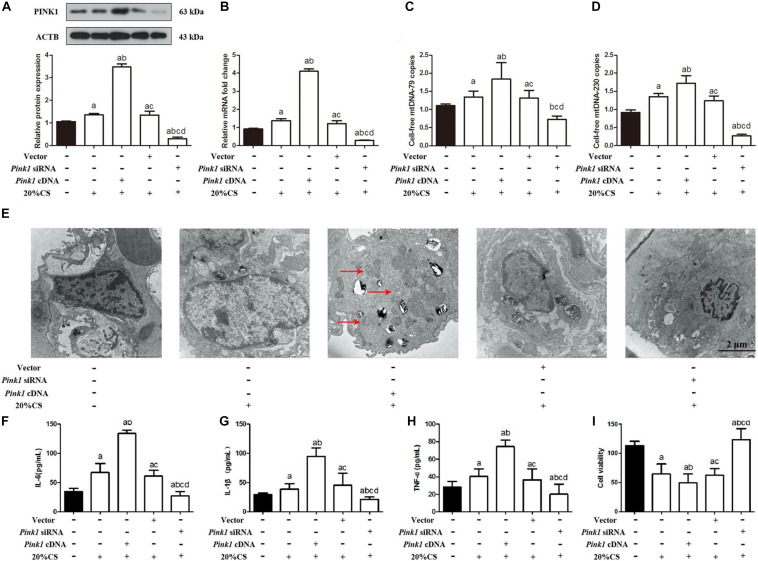
PINK1 promotes mitophagy, which releases mtDNA that helps drive cyclic stretching-induced inflammation and injury. **(A,B)** Lung epithelial cells were treated with *Pink1* siRNA, cDNA or empty vector and exposed to cyclic stretching (CS) at 20% tension for 4 h. As expected, Western blot and RT-qPCR showed decreased PINK1 protein and mRNA expression in *Pink1*-deficient lung epithelial cells, but increased PINK expression in cells treated with *Pink1* cDNA. **(C)** RT-qPCR was simultaneously performed to assess the cell-free mtDNA-79 copies. **(D)** RT-qPCR was simultaneously performed to assess the cell-free mtDNA-230 copies. **(E)** Transmission electron microscopy was performed to assess cell injury ultrastructurally (magnification ×20000). Red arrows indicate the autophagosomes. **(F–H)** Enzyme-linked immunosorbent assays were used to assess the levels of IL-1β, IL-6, and TNF-α in the culture medium. **(I)** MTT assay was used to examine the viability of cells. Experiments were performed in triplicate. ^a^*P* < 0.05 vs. control group; ^b^*P* < 0.05 vs. 20% CS group; ^c^*P* < 0.05 vs. *Pink1* cDNA + 20% CS group; ^d^*P* < 0.05 vs. *Pink1* empty vector + 20% CS group.

To explore the potential role of PINK1 in mediating mitophagy-induced mtDNA release, we transfected A549 cells with lentivirus encoding *Pink1* cDNA or anti-*Pink1* siRNA and confirmed that the respective cultures over- or under-expressed PINK1 protein and mRNA (Figures 2A,B and Supplementary Figure S2A). Then we subjected these cultures to CS. *Pink1* overexpression led to higher mtDNA release, while knockdown led to smaller releases (Figures 2C,D). Transmission electron microscopy showed higher cell injury in the presence of *Pink1* over-expression but a milder injury in the case of *Pink1* knockdown relative to cells expressing normal levels of endogenous PINK1 (Figure 2E). *Pink1* over-expression also led to higher secretion of IL-1β, IL-6, and TNF-α into the culture medium, while *Pink1* knockdown led to lower secretion relative to cells expressing normal levels of endogenous PINK1 (Figures 2F–H).

*Pink1* over-expression also decreased cell viability (Figure 2I), and then induced cell apoptosis (Supplementary Figures S2B–D), and reduced ATP levels (Supplementary Figure S2E) and ΔψM (Supplementary Figure S2F), while *Pink1* knockdown showed the conversed results relative to cells expressing normal levels of endogenous PINK1. Furthermore, A549 cells treated with PINK1 cDNA or siRNA showed the *Pink1* over-expression and knocking down, and the expression of PINK1 between A549 cells treated with PBS as control and A549 cells treated with vector was similar (Supplementary Figure S3A). There were no statistical differences noted on the expression of MAP1LC3B-II, BECN, SQSTM1 (Supplementary Figure S3B), cell injury (Supplementary Figure S3F); secretion of IL-1β, IL-6, and TNF-α (Supplementary Figures S3G–I); and the cell-free mtDNA-79 and mtDNA-230 copies (Supplementary Figures S3J,K) between A549 cells with the treatment of PINK1 cDNA or siRNA and vector and control A549 cells. A549 cells with the treatment of PINK1 cDNA or siRNA and vector both induced the mild increase of TLR9 and NF-κB in comparison to control A549 cells. It suggested that *Pink1* over-expression and knocking down did not affect the protein levels of TLR9 and NF-κB directly, and PINK1 did not interact with TLR9, MyD88, and/or NF-κB influencing their phosphorylation status and eventually their stabilization or activation. These results suggested that PINK1 may help drive mitophagy-induced mtDNA release in VILI.

### *Pink1* Knockdown in Lung Epithelial Cells Attenuates Activation of Mitophagy and Inhibits the TLR9/MyD88 Pathway

**FIGURE 3:**
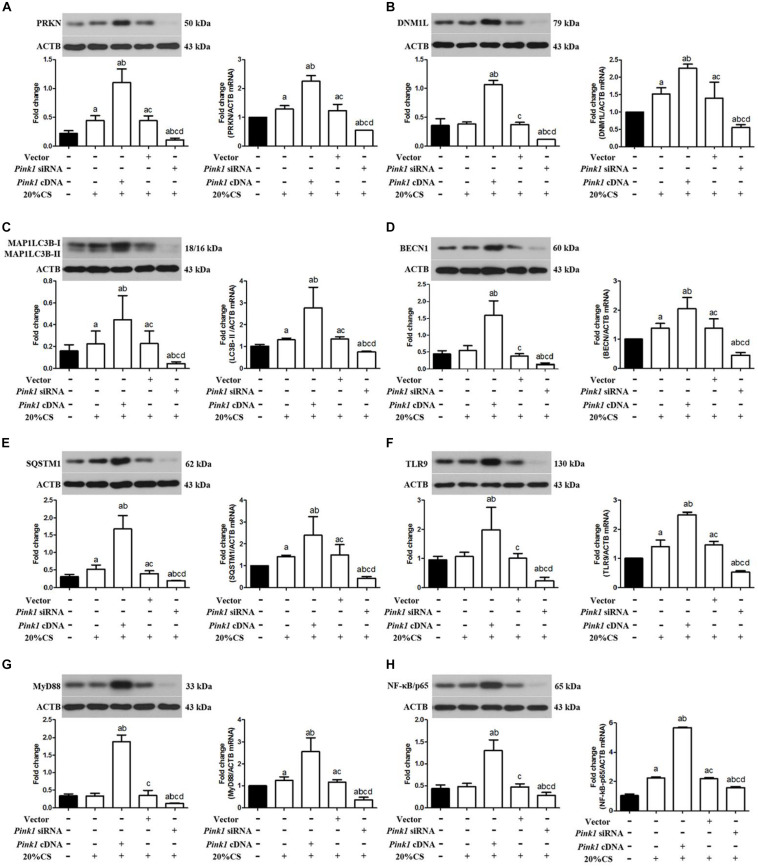
Up-regulation of PINK1 increases activation of mitophagy and TLR9/MyD88 signaling in cyclic stretching-induced inflammation and injury. Lung epithelial cells were treated with *Pink1* siRNA, cDNA or empty vector and exposed to cyclic stretching (CS) at 20% tension for 4 h. Western blots and RT-qPCR were performed to assay expression of **(A)** PRKN, **(B)** DNM1L, **(C)** MAP1LC3B, **(D)** BECN1, **(E)** SQSTM1, **(F)** TLR9, **(G)** MyD88, and **(H)** p-NF-κB/p65. Experiments were performed in triplicate. ^a^*P* < 0.05 vs. control group; ^b^*P* < 0.05 vs. 20% CS group; ^c^*P* < 0.05 vs. *Pink1* cDNA + 20% CS group; ^d^*P* < 0.05 vs. *Pink1* empty vector + 20% CS group.

To determine whether PINK1 may drive mitophagy-induced mtDNA release by stimulating mitophagy, we assayed the expression of PRKN, DNM1L, and autophagy-related proteins including BECN1, MAP1LC3B-II, and SQSTM1 in the presence of *Pink1* over-expression or knockdown following CS. *Pink1* over-expression increased levels of PRKN, DNM1L, MAP1LC3B-II, BECN1, and SQSTM1 protein and mRNA (Figures 3A–E), but *Pink1* knockdown triggered the opposite effects. These results suggested that PINK1 may help drive mitophagy induced by VILI.

To elucidate how PINK1 may regulate mitophagy, we asked whether it may affect the TLR9/MyD88 pathway, since *Pink1* mediated mtDNA release (Klionsky et al., 2016). Compared to normal levels of endogenous PINK1, cells over-expressing *Pink1* showed higher levels of TLR9, MyD88, and NF-κB/p65 protein and mRNA, while cells under-expressing *Pink1* produced lower levels of all three proteins (Figures 3F–H). These results suggested that PINK1 may help drive ventilation-induced cell injury and inflammation by activating mitophagy via the TLR9/MyD88 pathway and that inhibiting PINK1 may have therapeutic efficacy against VILI.

### Released mtDNA Activates the TLR9/MyD88 Pathway to Drive CS-Induced Inflammation and Cell Injury

**FIGURE 4:**
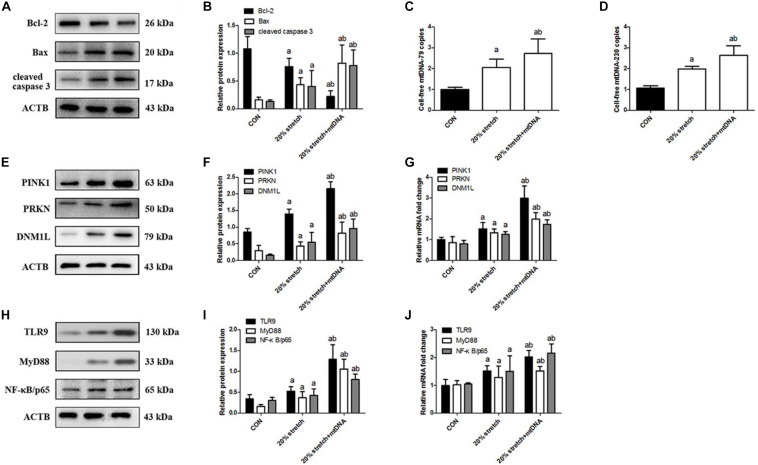
Released mtDNA targets the TLR9/MyD88 pathway affecting mitophagy in cyclic stretching (CS)-induced inflammation and injury. Lung epithelial cells were treated with exogenous mtDNA or an equal volume of phosphate buffer and exposed to CS at 20% tension for 4 h. These cells exposed to physiological stretching (5% for 4.0 h) were applied in the CON group. **(A,B)** Western blotting was performed to determine the expression of the apoptotic protein of Bcl-2, Bax, and cleaved caspase 3. **(C)** RT-qPCR was simultaneously performed to assess the cell-free mtDNA-79 copies. **(D)** RT-qPCR was simultaneously performed to assess the cell-free mtDNA-230 copies. **(E,F)** Western blotting was performed to determine the protein expression of PINK1, PRKN, and DNM1L. **(G)** RT-qPCR was simultaneously performed to assess mRNA levels of PINK1, PRKN, and DNM1L. **(H,I)** Western blotting was performed to determine the protein expression of TLR9, MyD88, and NF-κB/p65. **(J)** RT-qPCR was simultaneously performed to assess mRNA levels of TLR9, MyD88, and NF-κB/p65. Experiments were performed in triplicate. ^a^*P* < 0.05 vs. control group; ^b^*P* < 0.05 vs. 20% CS group.

To examine whether mtDNA directly activates stretching-induced inflammation and cell injury, A549 cells were pretreated with exogenous mtDNA and then subjected to CS at 20% tension. Addition of mtDNA aggravated CS-induced cell injury (Supplementary Figure S4A) and production of IL-1β, IL-6, and TNF-α (Supplementary Figures S4B–D) compared with cells receiving CS at 20% tension alone, simultaneously showing decreased cell viability (Supplementary Figure S4E), ATP levels (Supplementary Figure S4F), and ΔψM (Supplementary Figure S4G). Then, the addition of mtDNA increased A549 cell apoptosis (Figures 4A,B) and induced a higher level of mtDNA (Figures 4C,D). These effects were due to an effect of mtDNA on mitophagy since pre-treatment with mtDNA increased the expression of PINK1, PRKN, and DNM1L (Figures 4E–G).

Since mtDNA is a ligand of TLR9 (Dai et al., 2015; Lin et al., 2018), we next investigated whether mtDNA activates the TLR9/MyD88 pathway. Indeed, treatment with mtDNA up-regulated TLR9, MyD88, and NF-κB/p65 in A549 cells receiving CS at 20% tension compared with cells receiving CS at 20% tension alone (Figures 4H–J). These results suggested that released mtDNA activates the TLR9/MyD88 pathway in lung epithelial cells in response to CS.

### TLR9 Helps Drive CS-Induced Lung Epithelial Cell Injury by Up-Regulating MyD88 and NF-κB/p65

**FIGURE 5:**
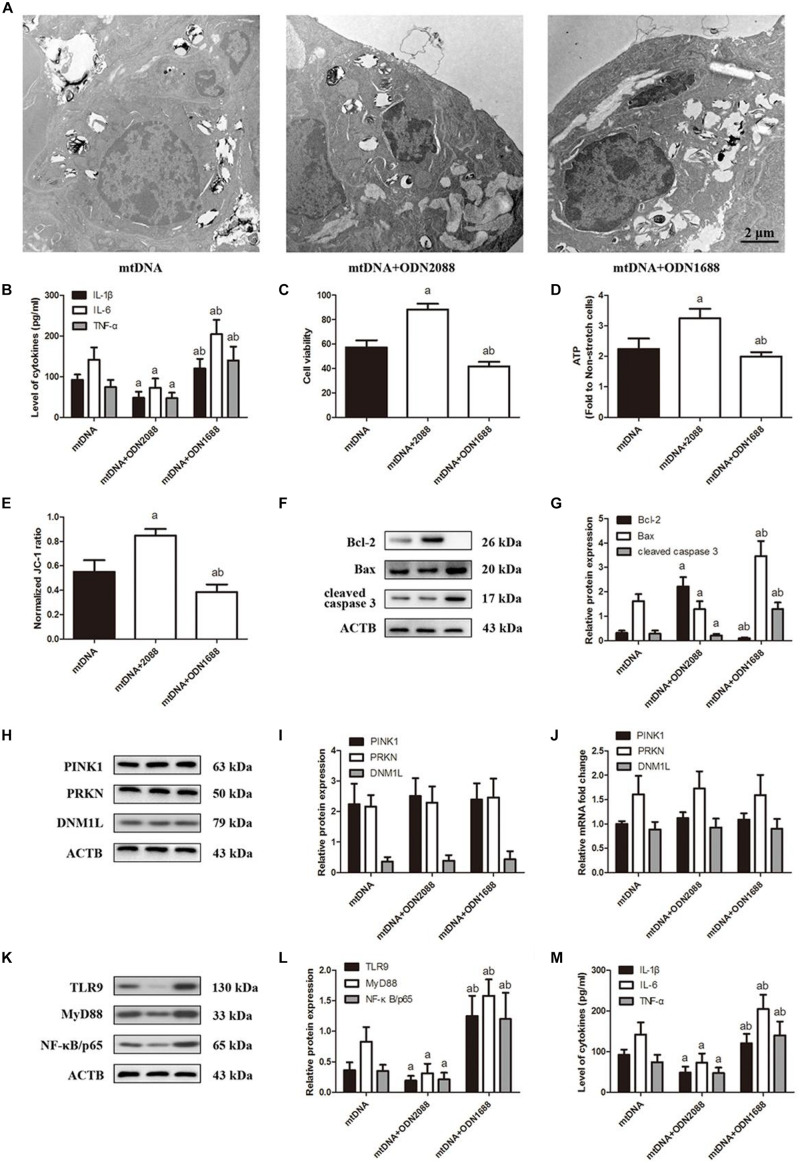
TLR9 down-regulation ameliorates cyclic stretching-induced inflammation response and injury. Lung epithelial cells were treated with TLR9 receptor antagonist ODN2088 or TLR9 receptor agonist ODN1668 and exposed to cyclic stretching (CS) at 20% tension for 4 h. **(A)** Transmission electron microscopy was performed to assess cell injury ultrastructurally (magnification ×20000). **(B)** Enzyme-linked immunosorbent assays were used to assess the level of IL-1β, IL-6, and TNF-α in the culture medium. **(C)** MTT assay was used to examine the viability of cells. **(D)** ATP determination assay kit was used to assess the ATP level of cells. **(E)** Mitochondrial membrane potential assay kit was used to assess the mitochondrial membrane potential level of cells. **(F,G)** Western blotting was performed to determine the expression of the apoptotic protein of Bcl-2, Bax, and cleaved caspase 3. **(H,I)** Western blotting was performed to determine the protein expression of PINK1, PRKN, and DNM1L. **(J)** RT-qPCR was simultaneously performed to assess mRNA levels of PINK1, PRKN, and DNM1L. **(K,L)** Western blotting was performed to determine the protein expression of TLR9, MyD88, and NF-κB/p65. **(M)** RT-qPCR was simultaneously performed to assess mRNA levels of TLR9, MyD88, and NF-κB/p65. Experiments were performed in triplicate. ^a^*P* < 0.05 vs. control group; ^b^*P* < 0.05 vs. ODN2088 group.

To investigate the association between TLR9 expression and CS-induced cell injury, we pretreated lung epithelial cells with the TLR9 antagonist ODN2088 or agonist ODN1668 then exposed them to CS. ODN2088 attenuated stretching-induced cell injury (Figure 5A) and production of IL-1β, IL-6, and TNF-α (Figure 5B), while ODN1668 aggravated it. ODN2088 also increased cell viability (Figure 5C), ATP production (Figure 5D), and ΔψM (Figure 5E) as well as attenuated cell apoptosis (Figures 5F–G), while ODN1688 triggered the opposite effects. These effects were not due to an effect of TLR9 on mitophagy since pre-treatment with ODN2088 or ODN1688 both did not affect the expression of PINK1, PRKN, and DNM1L (Figures 5H–J). ODN2088 significantly down-regulated TLR9 while ODN1668 up-regulated it. These changes in TLR9 levels led to the same changes in MyD88 and NF-κB/p65 (Figures 5K–M). These results indicated that TLR9 can promote CS-induced injury of lung epithelial cells by activating MyD88 and NF-κB/p65.

### MyD88 Helps Drive CS-Induced Lung Epithelial Cell Injury by Up-Regulating NF-κB/p65

**FIGURE 6:**
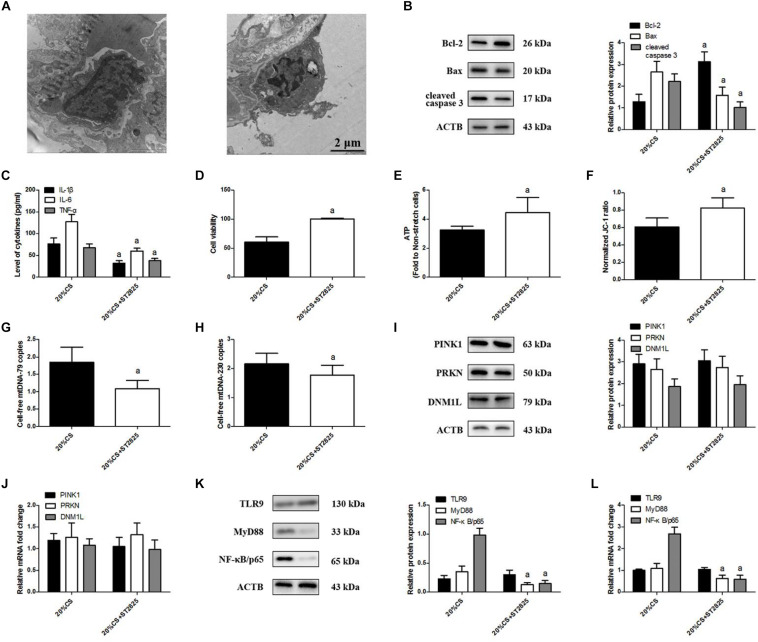
MyD88 down-regulation ameliorates cyclic stretching (CS)-induced inflammation and injury. Lung epithelial cells were treated with the MyD88 inhibitor ST2825 or not and exposed to CS at 20% tension for 4 h. **(A)** Transmission electron microscopy was performed to assess cell injury ultrastructurally (magnification ×20000). **(B)** Western blotting was performed to determine the expression of the apoptotic protein of Bcl-2, Bax, and cleaved caspase 3. **(C)** Enzyme-linked immunosorbent assays were used to assess the levels of IL-1β, IL-6, and TNF-α in the culture medium. **(D)** MTT assay was used to examine the viability of cells. **(E)** ATP determination assay kit was used to assess the ATP level of cells. **(F)** Mitochondrial membrane potential assay kit was used to assess the mitochondrial membrane potential level of cells. **(G)** RT-qPCR was simultaneously performed to assess the cell-free mtDNA-79 copies. **(H)** RT-qPCR was simultaneously performed to assess the cell-free mtDNA-230 copies. **(I)** Western blotting was performed to determine the protein expression of PINK1, PRKN, and DNM1L. **(J)** RT-qPCR was simultaneously performed to assess mRNA levels of PINK1, PRKN, and DNM1L. **(K)** Western blotting was performed to determine the protein expression of TLR9, MyD88, and NF-κB/p65. **(L)** RT-qPCR was simultaneously performed to assess mRNA levels of TLR9, MyD88, and NF-κB/p65. Experiments were performed in triplicate. ^a^*P* < 0.05 vs. control group.

To elucidate the effects of MyD88 on mtDNA release and TLR9 signaling, we pretreated lung epithelial cells with ST2825 then exposed them to CS at 20% tension. ST2825 largely attenuated stretching-induced cell injury (Figure 6A); cell apoptosis (Figure 6B); and production of IL-1β, IL-6, and TNF-α (Figure 6C) as well as impaired cell viability (Figure 6D) and increased ATP production (Figure 6E) and ΔψM (Figure 6F), and these changes correlated with the reduction of cell-free mtDNA release (Figures 6G,H). ST2825 triggered the down-regulation of MyD88 and NF-κB/p65. At the same time, ST2825 did not alter the levels of PINK1, PRKN, DNM1L (Figures 6I,J), and TLR9 protein or mRNA (Figures 6K,L). These results indicate that MyD88 can promote stretching-induced injury in lung epithelial cells by activating NF-κB/p65 independently of mitophagy and TLR9 activation.

## Discussion

Here we provide evidence for an important role of PINK1 in driving mitophagy, which releases mtDNA, which in turn activates the TLR9/MyD88 pathway in a cell culture model of VILI. These results are consistent with our previous findings in rats that PINK1-mediated mitophagy releases mtDNA via the TLR9/MyD88 pathway to drive mechanical cell injury (Lin et al., 2018). The present study is the first to provide direct evidence that CS-induced mitophagy contributes to mtDNA release and subsequent inflammation, and it begins to clarify the molecular pathways involved.

We found that CS at 20% tension triggered PINK1/PRKN-mediated mitophagy. Mechanical ventilation has been demonstrated to induce the oxidative damage of mitochondria, up-regulating the high-mobility group box1 and nuclear factor-erythroid 2-related factor 2 (Dong et al., 2015; de Souza et al., 2018). Such mitophagy is activated to eliminate damaged mitochondria, leading to the accumulation of reactive oxygen species and a decrease in ΔψM (Hou et al., 2018; Lin et al., 2018). This mitophagy leads to mtDNA release (Oka et al., 2012), as we confirmed in the present study, and this mtDNA acts as a DAMP to stimulate the immune response and activate neutrophils via a TLR9-dependent pathway (Zhang et al., 2010; Schafer et al., 2016).

Our results showed that mtDNA did not activate inflammation or cell injury in PINK1-deficient cells. This suggests that PINK1 may be the key driver of mitophagy induced by VILI. Indeed, PINK1 as well as the E3 ubiquitin (ligase Parkin) have been identified as key regulators of mitophagy in other cellular contexts (Harper et al., 2018). Once mitochondria are damaged, PINK1 is stabilized on the outer mitochondrial membrane to phosphorylate PRKN and transform LC3B-I to LC3B-II to facilitate autophagosome formation (Harper et al., 2018; Wang et al., 2018). DNM1L as a mediator of mitochondrial priming is associated with PINK1/PRKN-mediated ubiquitination and mitophagy (Thangaraj et al., 2018). Consistent with these observations, we found that PINK1 knockdown down-regulated PRKN, DNM1L, LC3B-II, BECN1, and SQSTM1, while PINK1 over-expression led to the opposite results. These results indicated that PINK1-driven mitophagy releases mtDNA in response to CS.

Mitochondrial DNA is critical for mitochondrial functions and packaged by mtDNA-interacting proteins to form DNA protein nucleoids (Ingelsson et al., 2018). Jian et al. (2018) found that assembly machinery 50 links mitochondrial dynamics and mitophagy and that depletion induces the elimination of mitochondria without affecting mtDNA content. The cell-free mtDNA was significantly increased in A549 cells with PINK1 over-expression and mitophagy, suggesting that damaged mitophagy was degraded by various hydrolases in autolysosomes resulting in the disruption of the mitochondrial membrane and releases of mtDNA.

We focused on the TLR9/NF-κB/p38 MAPK pathway as the mechanism through which released mtDNA triggers inflammation (Oka et al., 2012; Feng et al., 2013). Released mtDNA can also cause inflammatory responses through NLRP3 and AIM2 inflammasome (Fernandes-Alnemri et al., 2009; Carlos et al., 2017), cGAS-cGAMP-STING pathway, and neutrophil extracellular traps (Hornung et al., 2009; McIlroy et al., 2014; White et al., 2014). Released cell-free mtDNA plays an important role in the immune and inflammatory response during severe trauma, non-hemolytic transfusion reaction, and ischemia-reperfusion injury (Hu et al., 2015; Timmermans et al., 2016; Simmons et al., 2017). A previous study (Hu et al., 2015) reported that mtDNA activates inflammation and accelerates the release of TNF-α, IL-6, and IL-10 during liver ischemia-reperfusion injury. Here we show that released mtDNA acts via the TLR9/MyD88 pathway to induce stretching-induced inflammation in lung epithelial cells. Previously we showed that TLR9-MyD88 signaling in alveolar macrophages contributes to VILI (Huang et al., 2017; Lin et al., 2018). Released mtDNA, like bacterial DNA, contains CpG motifs and therefore binds to TLR9 to activate pro-inflammatory signaling and regulate the expression of MyD88, activating NF-κB and stimulating the production of pro-inflammatory factors (Latz et al., 2004; Chao, 2009).

Interestingly, Bueno et al. (2019) recently found that A549 cells treated with tunicamycin to induce endoplasmic reticulum stress, which down-regulated the expression of PINK1, reducing mitophagy and increasing the susceptibility to lung fibrosis. However, their previous study demonstrated that impaired mitochondria in idiopathic pulmonary fibrosis and aging lungs with upregulation of endoplasmic reticulum stress were associated with low expression of PINK1, and PINK1 deficiency leads to swollen, dysfunctional mitochondria and defective mitophagy (Bueno et al., 2015). These results suggested that the role of PINK1 expression in idiopathic pulmonary fibrosis and aging lungs should be further identified. In the present study, we showed that the released mtDNA via PINK1-dependent mitophagy is necessary but not sufficient to activate the TLR9/MyD88 pathway and thereby induce inflammation in A549 cells undergoing CS exposure.

However, there was a major weakness of the study, which was a reliance on a single cell line, A549, for all the data. These data need to be confirmed in more relevant models using primary epithelial cells. TLR9 is localized to the endosomal membrane, senses CpG DNA, and activates the transcription factors NF-κB and interferon regulatory factor 7, in turn, leading to the expression of genes encoding proinflammatory cytokines and interferons, respectively. Hence, another limitation that was we did not identify the role of cGAS, AIM2, and interferon regulatory factor 7 in this study. Furthermore, we had some difficulties in collecting BALF from patients undergoing mechanical ventilation and identifying the role of released mtDNA via PINK1-dependent mitophagy in humans needing clinic support.

**FIGURE 7:**
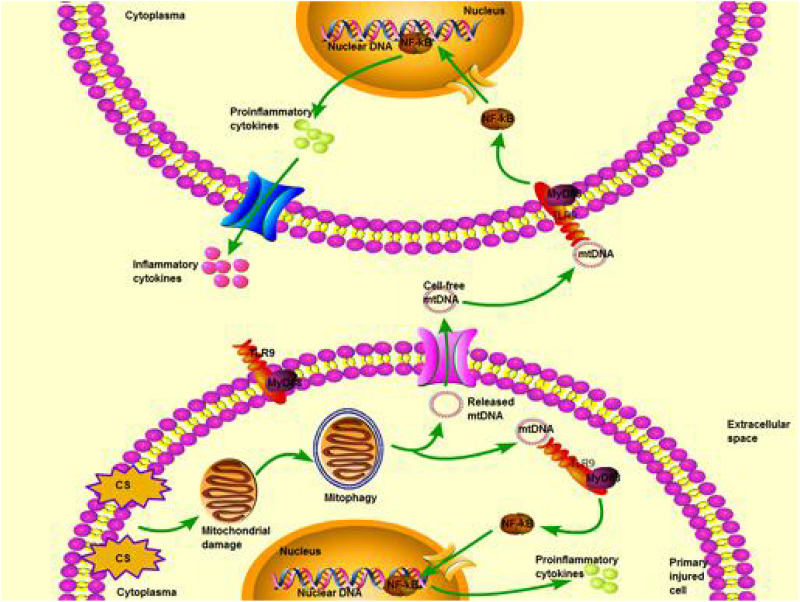
Schematic illustrating a potential pathway from cyclic stretching (CS) to inflammation and injury in lung epithelial cells via Toll-like receptor (TLR)-9 stimulation and release of mitochondrial DNA (mtDNA). Exposure of excessive CS triggers mitochondrial damage that is degraded by activated mitophagy. Subsequently, mtDNA is released from degraded mitochondria to extracellular fluid, then it is recognized and combined with TLR-9 in the other normal cells. TLR9 interacts with myeloid differentiation primary response 88 (MyD88) through downstream TLR9/MyD88 signaling. This pathway finally leads to activating nuclear factor kappa-light-chain-enhancer of activated B cells (NF-κB) and translation of proinflammatory cytokines. Proinflammatory cytokines are then matured and transformed into inflammatory cytokines for cascade amplification of inflammatory response and cell injury.

In conclusion, the present study in a cell culture model of VILI suggests that CS up-regulates PINK1 expression, which triggers mitophagy with mtDNA release, which activates the TLR9/MyD88 pathway and ultimately induces inflammation and cell injury. Our *in vitro* results lead us to propose PINK1-mediated mitophagy with mtDNA release as a therapeutic target via TLR9/MyD88 signaling in mechanical stretching-induced acute lung injury (Figure 7).

## Data Availability Statement

The raw data supporting the conclusions of this article will be made available by the authors, without undue reservation.

## Author Contributions

L-HP designed and directed the overall study. RJ and Z-KH carried out all the experiments and wrote the manuscript. RJ, FL, SH, S-SZ, HD, and W-YG collected and analyzed the data. All authors read and approved the final manuscript.

## Conflict of Interest

The authors declare that the research was conducted in the absence of any commercial or financial relationships that could be construed as a potential conflict of interest.

## Supplementary Material

FIGURE S1 Overstretching of lung epithelial cells triggers acute inflammatory cell injury with apoptosis, reduced ATP production, and mitochondrial membrane potential. Lung epithelial cells were challenged for 4 h with cyclic stretching (CS) at 5% or 20% tension or left without CS. **(A)** Transmission electron microscopy was performed to assess cell injury ultrastructurally (magnification ×20000). Red arrows indicate the autophagosomes. **(B–D)** Enzyme-linked immunosorbent assays were used to assess the level of IL-1β, IL-6, and TNF-α in the culture medium. **(E)** MTT assay was used to examine the viability of cells. **(F)** Western blotting was performed to determine the expression of the apoptotic protein of Bcl-2, Bax, and cleaved caspase 3. **(G)** ATP determination assay kit was used to assess the ATP level of cells. **(H)** Mitochondrial membrane potential assay kit was used to assess the mitochondrial membrane potential level of cells. Experiments were performed in triplicate. ^a^*P* < 0.05, compared with the lung epithelial cells without CS; ^b^*P* < 0.05, compared with the lung epithelial cells exposed to CS at 5% tension.

FIGURE S2 Up-regulation of PINK1 promotes cell apoptosis and reduces ATP production and mitochondrial membrane potential. Lung epithelial cells were treated with *Pink1* siRNA, cDNA or empty vector and exposed to cyclic stretching (CS) at 20% tension for 4 h. **(A)** Black-white and fluorescence micrographs of lung epithelial cells with or without transfection (magnification ×100). **(B–D)** Western blotting was performed to determine the expression of the apoptotic protein of Bcl-2, Bax, and cleaved caspase 3. **(E)** ATP determination assay kit was used to assess the ATP level of cells. **(F)** Mitochondrial membrane potential assay kit was used to assess the mitochondrial membrane potential level of cells. Experiments were performed in triplicate. ^a^*P* < 0.05 vs. control group; ^b^*P* < 0.05 vs. 20% CS group; ^c^*P* < 0.05 vs. *Pink1* cDNA + 20% CS group; ^d^*P* < 0.05 vs. *Pink1* empty vector + 20% CS group.

FIGURE S3 Regulation of PINK1 expression did not promote cell apoptosis and reduces ATP production and mitochondrial membrane potential. Lung epithelial cells were treated with *Pink1* siRNA, cDNA or empty vector and did not expose to cyclic stretching (CS). **(A)** Western blotting was performed to determine the protein expression of PINK1, PRKN, and DNM1L. **(B)** Western blotting was performed to determine the protein expression of BECN1, MAP1LC3B, and SQSTM1. **(C)** Western blotting was performed to determine the protein expression of TLR9, MyD88, and NF-κB/p65. **(D)** ATP determination assay kit was used to assess the ATP level of cells. **(E)** Mitochondrial membrane potential assay kit was used to assess the mitochondrial membrane potential level of cells. **(F)** Transmission electron microscopy was performed to assess cell injury ultrastructurally (magnification ×20000). **(G–I)** Enzyme-linked immunosorbent assays were used to assess the levels of IL-1β, IL-6, and TNF-α in the culture medium. **(J,K)** RT-qPCR was simultaneously performed to assess the cell-free mtDNA-79 copies and mtDNA-230 copies. Experiments were performed in triplicate. ^a^*P* < 0.05 vs. control group; ^b^*P* < 0.05 vs. vector group; ^c^*P* < 0.05 vs. PINK1 cDNA group.

FIGURE S4 Released mtDNA targets triggers cell injury and secretion of inflammatory factors, promotes cell apoptosis, and reduces ATP production and mitochondrial membrane potential in cyclic stretching (CS)-induced inflammation and injury. Lung epithelial cells were treated with exogenous mtDNA or an equal volume of phosphate buffer and exposed to CS at 20% tension for 4 h. These cells exposed to physiological stretching (5% for 4.0 h) were applied in the CON group. **(A)** Transmission electron microscopy was performed to assess cell injury ultrastructurally (magnification ×20000). **(B–D)** Enzyme-linked immunosorbent assays were used to assess the levels of IL-1β, IL-6, and TNF-α in the culture medium. **(E)** MTT assay was used to examine the viability of cells. **(F)** ATP determination assay kit was used to assess the ATP level of cells. **(G)** Mitochondrial membrane potential assay kit was used to assess the mitochondrial membrane potential level of cells. Experiments were performed in triplicate. ^a^*P* < 0.05 vs. control group; ^b^*P* < 0.05 vs. 20% CS group.

## Abbreviations

Δψ M: mitochondrial membrane potential
ACTB: β-actin
ATP: adenosine triphosphate
Bax: BCL2 associated X apoptosis regulator
Bcl-2: BCL2 apoptosis regulator
BECN: beclin 1
CS: cyclic stretch
DAMP: damage-associated molecular pattern
DMEM: Dulbecco’s modified Eagle’s medium
DNM1L: dynamin 1 like
ELISA: Enzyme-linked immunosorbent assays
FBS: fetal bovine serum
HRP: horseradish peroxidase
IL: interleukin
MAP1LC3B: microtubule-associated protein 1 light chain 3 β
mtDNA: mitochondrial DNA
MyD88: myeloid differentiation factor 88
NF- κ B: nuclear factor- κ B
ODN: oligodeoxyribonucleotides
PBS: phosphate-buffered saline
PINK1: Phosphatase and tensin homolog induced kinase 1
PRKN: parkin RBR E3 ubiquitin-protein ligase
RT-qPCR: real time-quantitative PCR
SQSTM1: sequestosome 1
TLR9: Toll-like receptor 9
TNF: tumor necrosis factor
VILI: ventilator-induced lung injury.
