# Supplementary figures and images for: Mitophagy-Mediated mtDNA Release Aggravates Stretching-Induced Inflammation and Lung Epithelial Cell Injury via the TLR9/MyD88/NF-κB Pathway
Source: Front Cell Dev Biol. 2020 Sep 4;8:819. doi: 10.3389/fcell.2020.00819 (PMC7504878; doi:10.3389/fcell.2020.00819)

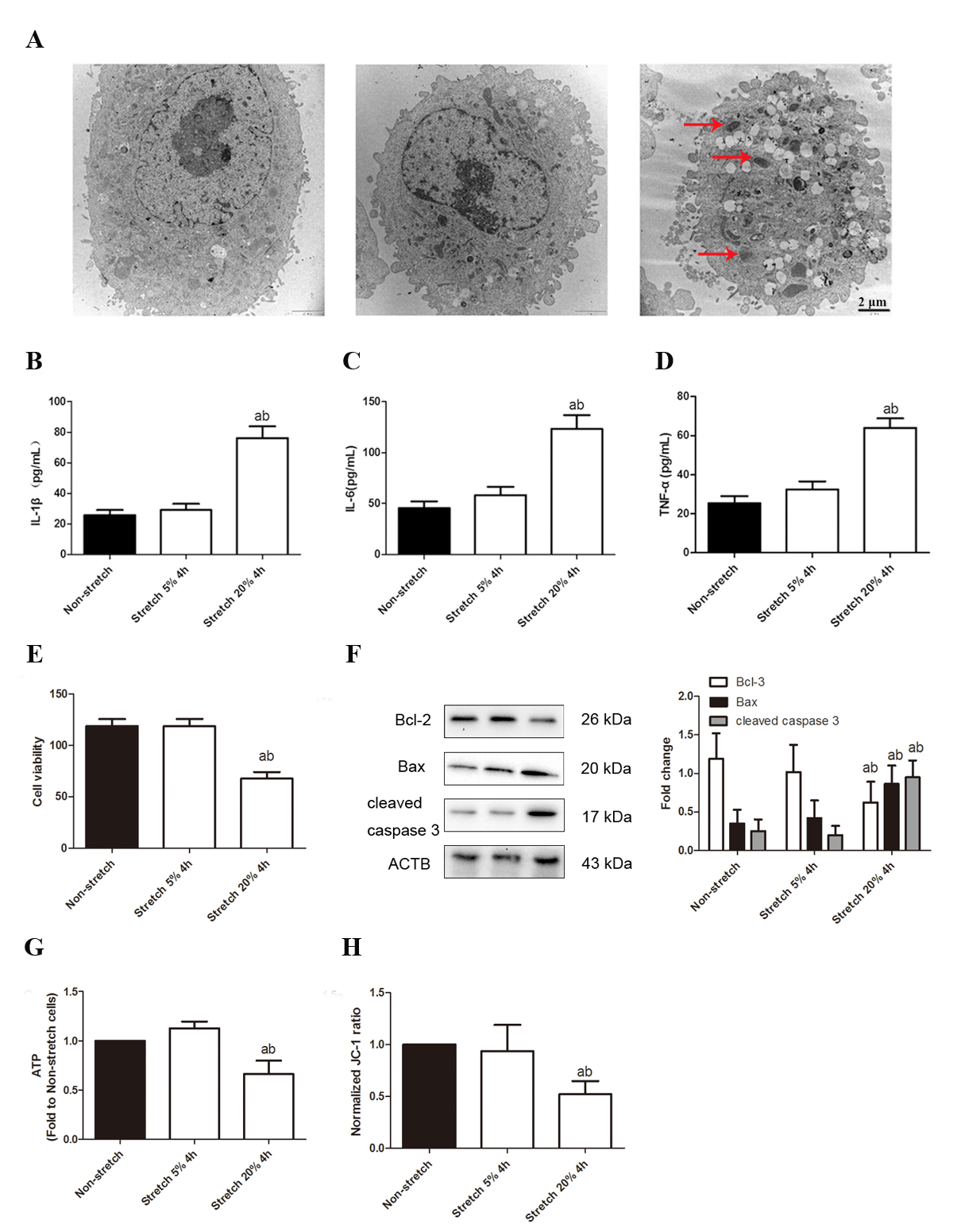

Supplement: FIGURE S1 — Overstretching of lung epithelial cells triggers acute inflammatory cell injury with apoptosis, reduced ATP production, and mitochondrial membrane potential. Lung epithelial cells were challenged for 4 h with cyclic stretching (CS) at 5% or 20% tension or left without CS. (A) Transmission electron microscopy was performed to assess cell injury ultrastructurally (magnification ×20000). Red arrows indicate the autophagosomes. (B–D) Enzyme-linked immunosorbent assays were used to assess the level of IL-1β, IL-6, and TNF-α in the culture medium. (E) MTT assay was used to examine the viability of cells. (F) Western blotting was performed to determine the expression of the apoptotic protein of Bcl-2, Bax, and cleaved caspase 3. (G) ATP determination assay kit was used to assess the ATP level of cells. (H) Mitochondrial membrane potential assay kit was used to assess the mitochondrial membrane potential level of cells. Experiments were performed in triplicate. aP < 0.05, compared with the lung epithelial cells without CS; bP < 0.05, compared with the lung epithelial cells exposed to CS at 5% tension. [file Image_1.TIF]

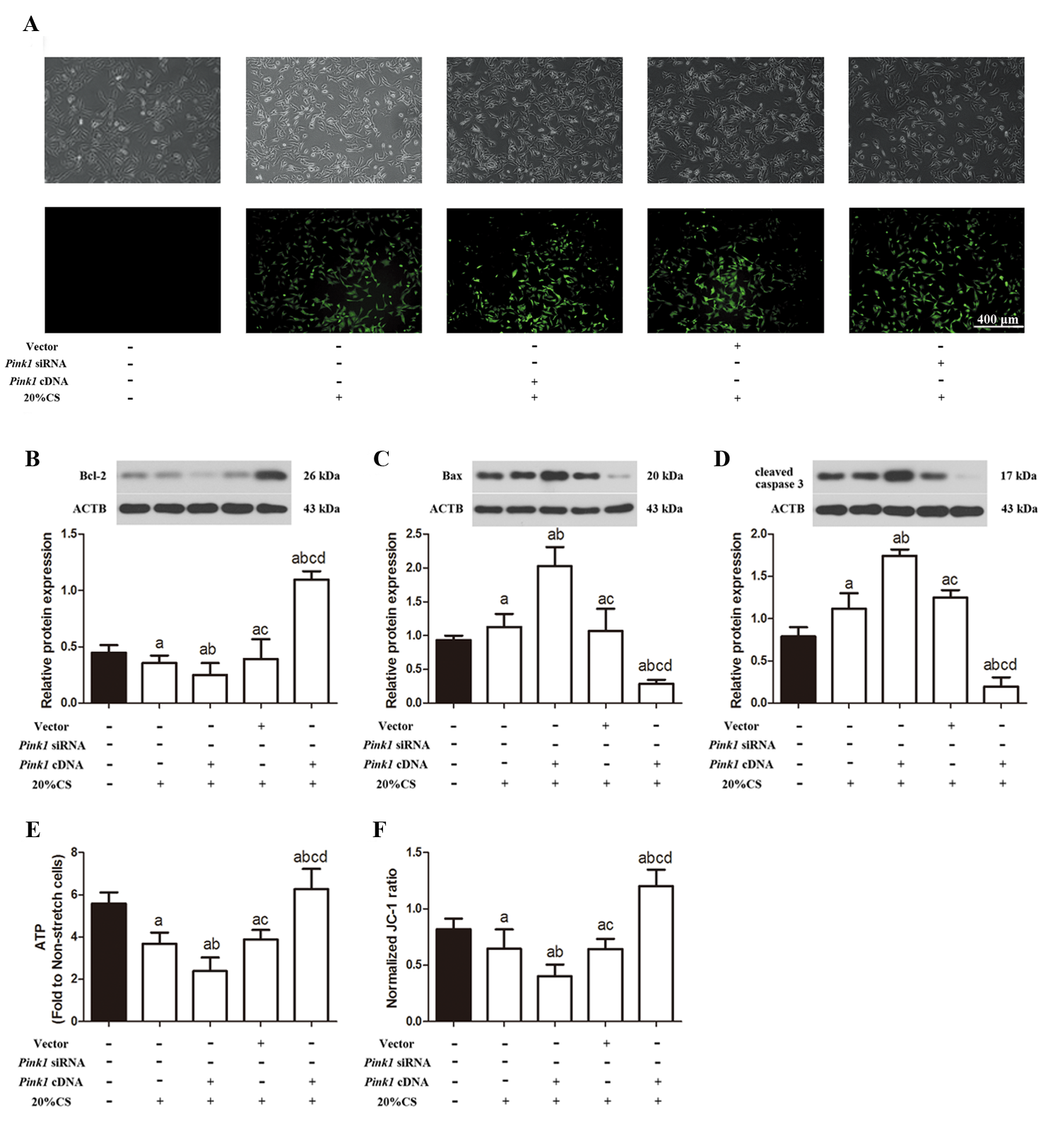

Supplement: FIGURE S2 — Up-regulation of PINK1 promotes cell apoptosis and reduces ATP production and mitochondrial membrane potential. Lung epithelial cells were treated with Pink1 siRNA, cDNA or empty vector and exposed to cyclic stretching (CS) at 20% tension for 4 h. (A) Black-white and fluorescence micrographs of lung epithelial cells with or without transfection (magnification ×100). (B–D) Western blotting was performed to determine the expression of the apoptotic protein of Bcl-2, Bax, and cleaved caspase 3. (E) ATP determination assay kit was used to assess the ATP level of cells. (F) Mitochondrial membrane potential assay kit was used to assess the mitochondrial membrane potential level of cells. Experiments were performed in triplicate. aP < 0.05 vs. control group; bP < 0.05 vs. 20% CS group; cP < 0.05 vs. Pink1 cDNA + 20% CS group; dP < 0.05 vs. Pink1 empty vector + 20% CS group. [file Image_2.TIF]

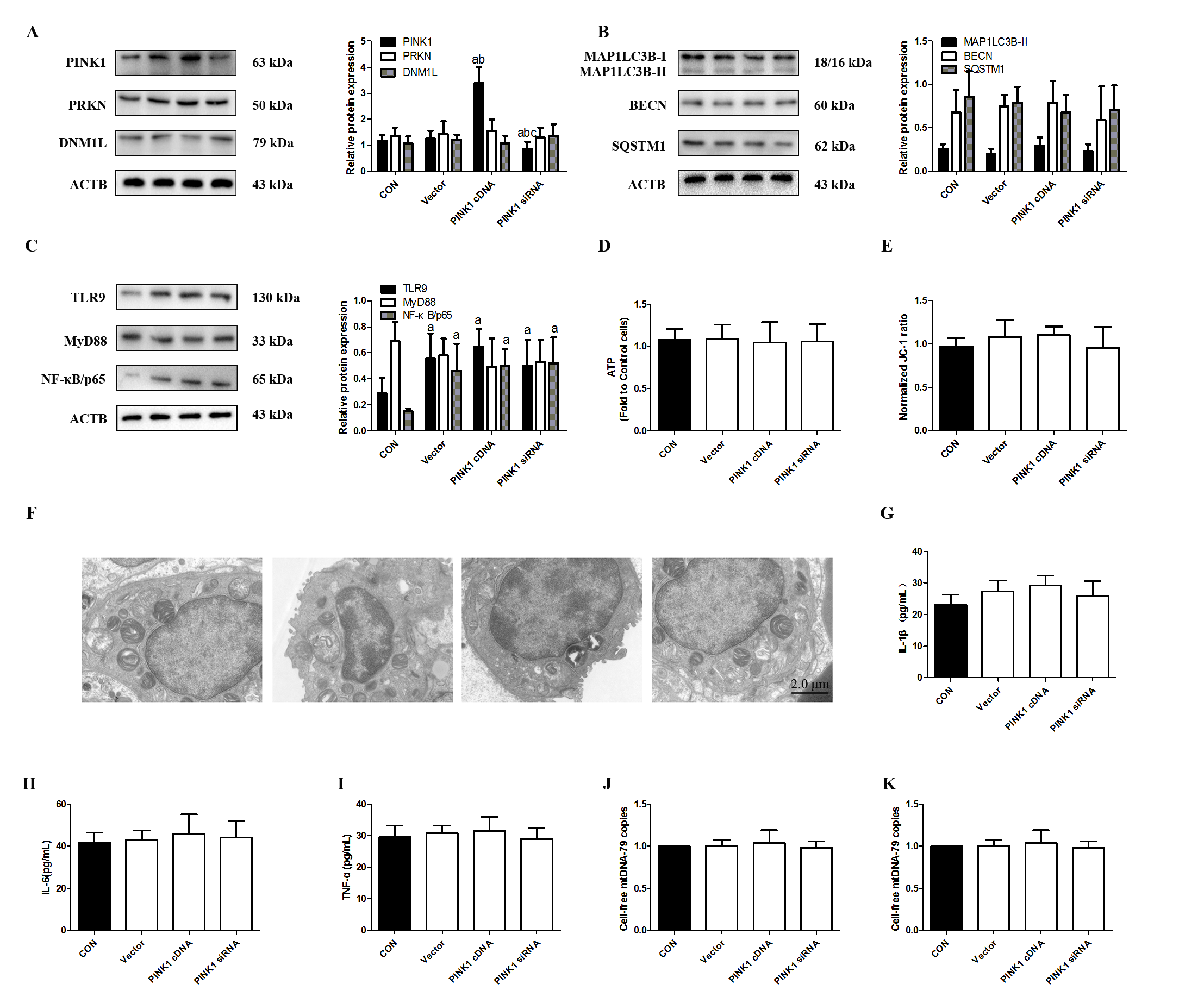

Supplement: FIGURE S3 — Regulation of PINK1 expression did not promote cell apoptosis and reduces ATP production and mitochondrial membrane potential. Lung epithelial cells were treated with Pink1 siRNA, cDNA or empty vector and did not expose to cyclic stretching (CS). (A) Western blotting was performed to determine the protein expression of PINK1, PRKN, and DNM1L. (B) Western blotting was performed to determine the protein expression of BECN1, MAP1LC3B, and SQSTM1. (C) Western blotting was performed to determine the protein expression of TLR9, MyD88, and NF-κB/p65. (D) ATP determination assay kit was used to assess the ATP level of cells. (E) Mitochondrial membrane potential assay kit was used to assess the mitochondrial membrane potential level of cells. (F) Transmission electron microscopy was performed to assess cell injury ultrastructurally (magnification ×20000). (G–I) Enzyme-linked immunosorbent assays were used to assess the levels of IL-1β, IL-6, and TNF-α in the culture medium. (J,K) RT-qPCR was simultaneously performed to assess the cell-free mtDNA-79 copies and mtDNA-230 copies. Experiments were performed in triplicate. aP < 0.05 vs. control group; bP < 0.05 vs. vector group; cP < 0.05 vs. PINK1 cDNA group. [file Image_3.TIF]

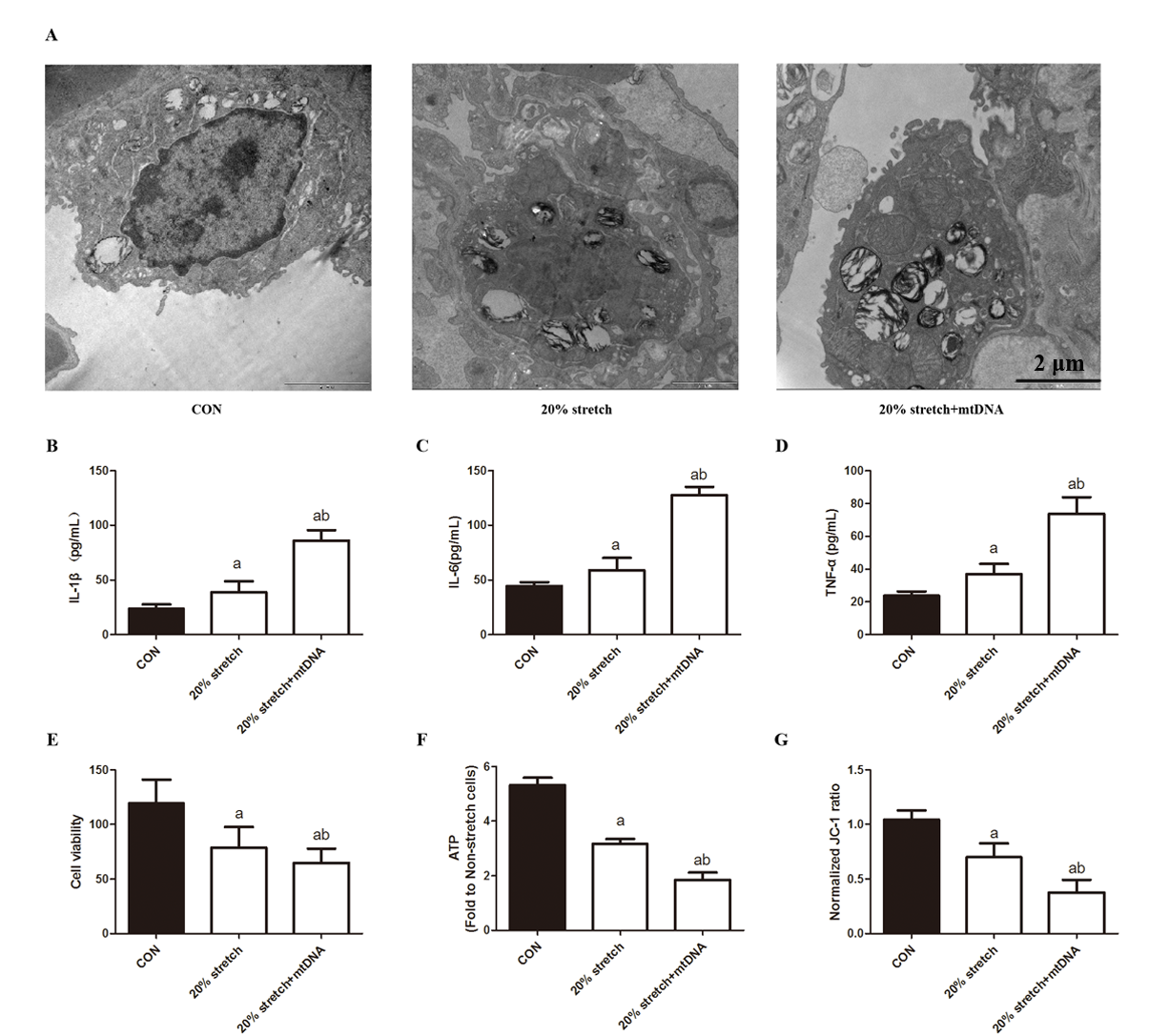

Supplement: FIGURE S4 — Released mtDNA targets triggers cell injury and secretion of inflammatory factors, promotes cell apoptosis, and reduces ATP production and mitochondrial membrane potential in cyclic stretching (CS)-induced inflammation and injury. Lung epithelial cells were treated with exogenous mtDNA or an equal volume of phosphate buffer and exposed to CS at 20% tension for 4 h. These cells exposed to physiological stretching (5% for 4.0 h) were applied in the CON group. (A) Transmission electron microscopy was performed to assess cell injury ultrastructurally (magnification ×20000). (B–D) Enzyme-linked immunosorbent assays were used to assess the levels of IL-1β, IL-6, and TNF-α in the culture medium. (E) MTT assay was used to examine the viability of cells. (F) ATP determination assay kit was used to assess the ATP level of cells. (G) Mitochondrial membrane potential assay kit was used to assess the mitochondrial membrane potential level of cells. Experiments were performed in triplicate. aP < 0.05 vs. control group; bP < 0.05 vs. 20% CS group. [file Image_4.TIF]
